# Cyclization Effects of Methoxy-Chalcones on Oxidative Stability: Impact on Biodiesel Additives

**DOI:** 10.1063/4.0000812

**Published:** 2025-10-27

**Authors:** Vitor Santos Duarte, Hamilton Barbosa Napolitano, Pal Perjesi

**Affiliations:** 1 Universidade Estadual de Goiás, Anápolis, Goiás, Brazil; 2 University of Pécs, Pécs, Baranya, Hungary

## Abstract

Biofuels are a cleaner alternative to fossil fuels, offering lower emissions and biodegradability. However, their low oxidative stability accelerates degradation, limiting broader application.^1^ Chalcones are organic compounds that can serve this function as additives, helping to enhance the oxidative stability of biodiesel and extend its shelf life, making it a more viable and durable energy source.^2^ In this study, we evaluated the effects of cyclization in four methoxy chalcones (MCH01–MCH04) to determine whether the cyclization of aromatic ring 1 influences their potential antioxidant activity (Figure 1). The molecules were synthesized via base-catalyzed Claisen-Schmidt condensation using appropriate aryl aldehydes.^3^ All compounds crystallized in the monoclinic crystal system *2_1_* with crystallographic structures achieving convergence values of R = 0.0385 (MCH01); 0.0455 (MCH02); 0.0220 (MCH03) and 0.0559 (MCH04). Molecular modeling was performed using theoretical analysis based on DFT, including frontier molecular orbitals, MEP map, QTAIM analysis, Hirshfeld surfaces and Fukui indices. The energy gap between the frontier molecular orbitals (HOMO-LUMO) for MCH01 was found to be 565 kJ/mol, which may be associated with its kinetic stability and reactivity. Oxidative stability tests on MCH01, when added to biodiesel, revealed an induction period of approximately 12.5 hours after 140 days of storage - exceeding the minimum commercial biodiesel requirement of 12 hours over a 60-day period - highlighting its antioxidant potential. A complete analysis of all samples is currently underway, and complementary tests will provide further insights into the cyclization effects of these molecules.
